# Impact of dynamic greenspace exposure on symptomatology in individuals with schizophrenia

**DOI:** 10.1371/journal.pone.0238498

**Published:** 2020-09-03

**Authors:** Philip Henson, John F. Pearson, Matcheri Keshavan, John Torous

**Affiliations:** 1 Department of Psychiatry, Beth Israel Deaconess Medical Center, Harvard Medical School, Boston, Massachusetts, United States of America; 2 Department of Anesthesiology, University of Utah, Salt Lake City, Utah, United States of America; Department of Psychiatry and Neuropsychology, Maastricht University Medical Center, NETHERLANDS

## Abstract

There are currently many tools available for capturing and defining the context of one’s environment. Digital phenotyping, the use of technology and sensors to capture moment-to-moment behavior, has shown potential in quantifying the lived experience of mental illness and in the identification of individualized targets related to recovery. Environmental data suggests that greenspace may have a restorative capacity on mental health. In this paper, we explore the relationship of greenspace derived from geolocation with self-reported symptomatology from individuals with schizophrenia as well as healthy controls. Individuals with schizophrenia had less exposure to greenspace than controls, but their exposure demonstrated a dosage effect: high greenspace environments were associated with lower symptoms for anxiety (Cohen’s d = -0.70), depression (d = -0.97), and psychosis (d = -0.94), whereas effect sizes for healthy controls were all negligible or small (d < 0.38). The notion that greenspace may have a more pronounced effect on individuals with mental illness presents both potential areas for recovery as well as implications for health care policy, especially in cities with a broad range of greenspace environments.

## Introduction

The impact of environmental exposure on the onset and course of schizophrenia remains challenging to quantify. Population based studies using static proxies for exposure like registered home address suggest that lack of greenspace exposure increases the risk of developing schizophrenia [1]. Yet the classic paradigm of urbanization and increased psychosis onset [2] has recently been challenged [3] and underscores the need for both more accurate and dynamic data on greenspace exposure in psychotic illnesses. In this paper, we explore the potential of digital phenotyping and geospatial analysis to inform new models of real-time associations between greenspace and symptoms.

Recent reviews have suggested that greenspace exposure is associated with improved mental health in both children and adults [4–6]. Theories for positive associations between mental health and greenspace include (1) reducing harm (e.g. reducing pollution associated with inflammation and onset of mental health conditions), (2) restoring capacities (e.g. improving attention and reducing stress [4]), and (3) building capacities (e.g. promoting physical activity and inducing social cohesion) [7, 8]. Neuroscience-based mechanisms of action remain an area of ongoing research, but there has been some evidence of greenspace exposure inducing improved functional and structural brain changes as well as activation of the parasympathetic nervous system [9]. Functional changes have been detected via electroencephalography (EEG) and extraction of frontal alpha asymmetry (FAA) values, which are often related to motivation and positive emotions [10]. Structural changes have been observed via brain magnetic resonance imaging (MRI), with positive associations found between lifelong exposure to greenspace and bilateral gray matter and white matter volumes [11]. While the underlying mechanism of action for greenspace impact on mental health likely involves elements from all three theories, the restorative capacities theory is a useful focus for studies of shorter duration as reducing harm and building capacities likely operate over many years.

Understanding the restorative capacities of greenspace on mental health requires estimating both the quantity and quality of greenspace exposure. While both variables matter, research suggests that quality may have a stronger association with mental health outcomes [12]. However, a challenge to this research is that static measures of greenspace exposure cannot account for the actual quantity or quality of exposure for any particular person. A recent review of 52 studies on greenspace and mental health noted the need for dynamic models of greenspace that utilize data for actual exposure, instead of theoretical exposure [13].

Smartphone digital phenotyping combined with analysis utilizing Geographic Information Systems (GIS) mapping software offers a practical method to estimate greenspace exposure in terms of both quantity and quality. Smartphone digital phenotyping using Global Navigation Satellite Systems (most commonly Assisted-Global Positioning System receivers, herein GPS) from a participant’s smartphone offers a feasible and practical means to estimate exposure to environments in a dynamic and real-time manner. This data can be analyzed with mapping software like Environmental Systems Research Institute’s (ESRI) ArcGIS which allows users to measure high level detail on greenspace quality and is an industry leading tool in transportation, urban planning, and environmental research. This exposure can be quantified in the Normalized Difference Vegetation Index (NDVI), a measure of greenness from NASA satellite data, which ranges between -1 to 1, with a threshold for “high” greenspace around 0.5–0.6 [14, 15]. Mental health researchers have already utilized NDVI with one study of 7,547 pregnant women in Bradford, UK reporting that those with higher quality greenspace exposure measured via NDVI were up to 23% less likely to report depressive symptoms [16]. Higher greenspace exposure measured by NDVI has also been associated with lower levels of stress [17] and is a mediating factor between neighborhood and mental health outcomes [18].

Combing smartphone digital phenotyping and greenspace exposure using ArcGIS with NDVI values offers the ability to explore the restorative capacities theory of greenspace on psychosis symptoms. Already, recent studies have used smartphone-based digital phenotyping to explore the dynamic relationship between mobility and mental health outcomes in schizophrenia. Depp et al. used smartphone-based GPS to follow 87 patients with schizophrenia over one week and noted that reduced mobility was associated with more negative symptoms although there was no association with positive symptoms, depression, or cognitive outcomes [19]. However, this study did not quantify greenspace exposure associated with mobility patterns. In this present study, we capture digital phenotyping data from 37 individuals with schizophrenia (SZ) and 26 healthy controls (HC) over three months to explore how greenspace exposure impacts self-reported symptoms. In line with the restorative hypothesis and prior work in depression and anxiety, we hypothesize that more exposure to greenspace will be associated with improved symptomatology, with a larger effect on patients than controls given that patients have a greater symptom burden and thus a greater potential for improvement via greenspace.

## Methods

This study was approved by the BIDMC IRB, Protocol #: 2017P000171, and written informed consent obtained from each subject. Thirty-seven participants diagnosed with schizophrenia in active outpatient treatment and 26 healthy controls partook in this three-month study. Individuals with schizophrenia were recruited from a community mental health center in Boston where they were in active treatment. Healthy controls were recruited from social media ads targeting the public and local area colleges. Geolocation was captured via smartphone GPS at a maximum rate of 1 Hz and contained a timestamp along with values for latitude, longitude, and accuracy. Self-reported symptoms were collected on the smartphone through an ecological momentary assessment (EMA) paradigm, which allowed for the completion of surveys remotely and with much more frequently than would be obtained from in-person clinical visits. Participants were not paid to complete surveys but were prompted to provide responses three times each week to questions about anxiety, depression, sleep, sociability, and psychotic symptoms. Each EMA survey collected consisted of a timestamp, response duration for each question, and score for each question. All individual question scores ranged from 0–3, representing “Not at all,” “Several times,” “More than half the time,” and “Nearly all the time.” All GPS and EMA data were stored securely in a HIPAA-compliant server maintained by our team. These data may be considered individually identifiable and can be shared only upon request.

Of the 90 participants who enrolled in the study, 63 participants (37 SZ and 26 HC) provided GPS within the established accuracy threshold of 50 meters along with concurrent self-reported survey data. Demographics of both patients and controls partaking in the study are presented below in Table 1.

**Table 1:** Participant demographics.

	HC (n = 26)	SZ (n = 37)	p
Age	30.04 (13.41)	37.75 (14.05)	0.038
Gender			0.550
Male	13 (50.0%)	19 (51.4%)	
Female	12 (46.2%)	14 (37.8%)	
Other	1 (3.8%)	4 (10.8%)	
Race			<0.001
American Indian or Alaskan Native	0 (0.0%)	3 (8.6%)	
Asian	20 (76.9%)	1 (2.9%)	
Black or African American	2 (7.7%)	11 (31.4%)	
Multiracial or Other	1 (3.8%)	1 (2.9%)	
White Caucasian	3 (11.5%)	19 (54.3%)	
Education			0.004
4-year college graduate or higher	21 (80.8%)	13 (35.1%)	
High school graduate/GED	3 (11.5%)	10 (27.0%)	
Some college	2 (7.7%)	12 (32.4%)	
Some high school	0 (0.0%)	2 (5.4%)	

63 smartphone-owning study participants from the greater Boston area participated in this three-month smartphone study.

### Geographic exposure, effect modifiers, confounders and GIS analysis

We performed a geospatial analysis of participant geographic exposures by importing time-stamped GPS locations from participant smartphones into a geodatabase in ArcGIS version 10.7.1 (ESRI, Redlands, CA). For this study, we limited analysis of participant exposures to the geographic extent of the Commonwealth of Massachusetts. We estimated geographic exposures using GPS location traces for the 3 hours prior to any EMA survey. We further eliminated all GPS trace points of greater than 50 meter in accuracy.

**Fig 1:**
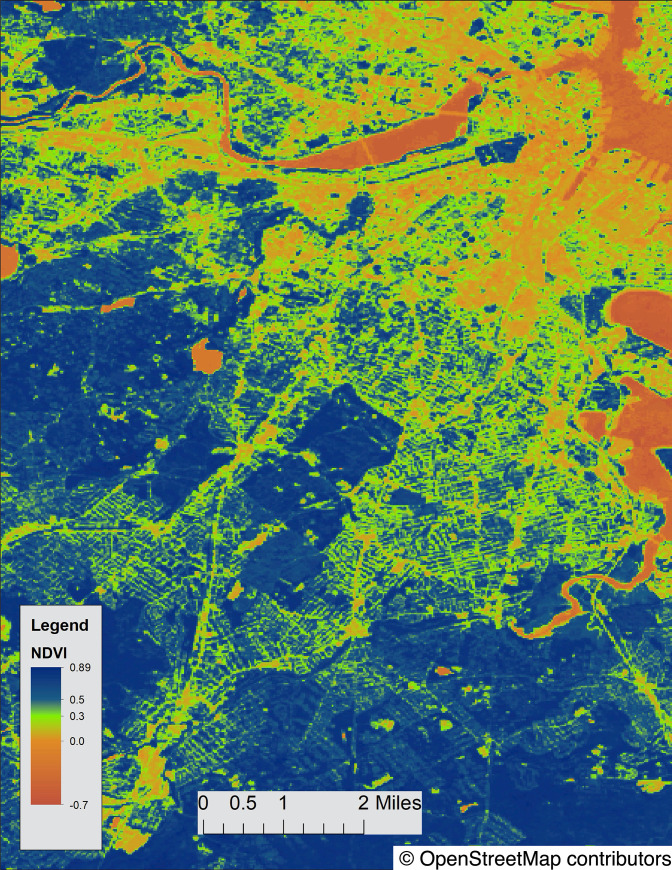
NDVI map of Boston, MA. NDVI values in Boston range from -0.7 to 0.89. Map data is available under the Open Data Commons Open Database License: https://www.openstreetmap.org/copyright.

Exposure to vegetation, as a proxy for greenspace, was estimated using data from the Moderate-resolution Imaging Spectroradiometer (MODIS), from NASA’s Terra satellite. This satellite-based vegetation index, or NDVI, calculated the difference in reflectance of leaves and chlorophyll to derive a measure of greenness ranging from -1.0 to 1.0, with higher values indicating greater levels of vegetative density (Fig 1) [20]. For this study, we utilized seasonal MODIS data of 30-meter resolution, which was taken on a quarterly basis. GPS timestamps were matched to those quarters (January 1, April 1, July 1, October 1) closest to the timestamp. Exposures to NDVI values greater than or equal to 0.5 were considered “high greenspace” while those less than 0.5 were considered “low greenspace.”

As geographic effect modifiers, we utilized population density data from the Massachusetts Bureau of Geographic Information (MassGIS) [21] at the Block Group level. For this study, we assessed the mean population density exposure for the duration of the GPS trace prior to the EMA. In addition, we utilized Per Capita Income from MassGIS as a geographic confounder, utilizing the same methodology of the 3-hour GPS trace.

We calculated the mean and standard deviation of NDVI for SZ and HC and assessed difference between the two population means with a two-sided t-test. Within each diagnostic group, we computed Pearson correlations at a 95% confidence level between NDVI and symptom scores to identify any associations between greenspace and symptomatology. High and low greenspace exposure groups were then created using an NDVI threshold of 0.5. To determine the magnitude of the effect of NDVI group on symptoms, we determined effect size using Cohen’s d in addition to performing a two-sided t-test. All analyses controlled for age, sex, and income of each participant as well as population density and neighborhood income related to their address.

## Results

Individuals with schizophrenia had lower exposure to greenspace than controls even after adjusting for exposure to neighborhood income and population density. The mean NDVI score for those with schizophrenia was 0.167 (SD = 0.03) and for healthy controls was 0.194 (SD = 0.02). The t-test rejected the null hypothesis revealing that the mean NDVI scores were statistically different between SZ and HC (p<0.001).

**Fig 2:**
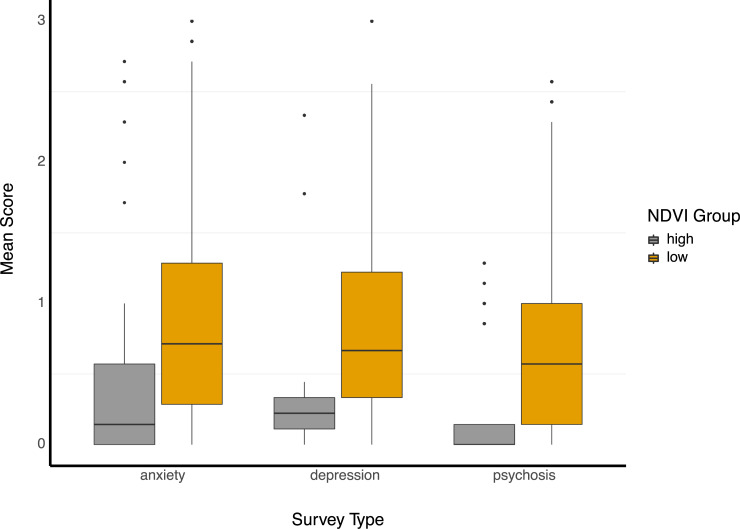
NDVI group differences. Box and whisker plots of symptom survey response (ranging from 0 to 3) separated by NDVI group. The box limits represent the 25th and 75th percentiles and a median line is drawn within. The whiskers extend to at most 1.5 times the inter-quartile range in each direction and outliers are plotted individually.

Correlation analysis revealed that more greenspace was associated with better symptom scores in every survey domain (anxiety, depression, psychosis, sleep, sociability). Coefficients were all low but significant (p < 0.001) and ranged from 0.007 (social, SZ) to 0.180 (depression, HC). After NDVI group separation of SZ, however, associations were much stronger with individuals in high NDVI settings exhibiting significantly lower symptoms for anxiety (d = -0.70, p<0.001), depression (d = -0.97, p<0.001), and psychosis (d = -0.94, p<0.001) than those from the low NDVI group (Fig 2). SZ in high NDVI settings also reported better sleep (d = -0.54, p<0.001) but worse levels of sociability (d = 0.55, p<0.001). Effect sizes for controls were all negligible or small, with anxiety exhibiting the largest effect size between the high NDVI group and low NDVI group (d = -0.38, p<0.001).

## Discussion

Measuring dynamic greenspace exposure in patients with schizophrenia via smartphone digital phenotyping, our results suggest a dosage effect with higher exposure associated with lower self-reported symptoms in only patients and not controls. This supports the restorative hypothesis as those with a mental health diagnosis have more to gain from greenspace exposure in terms of potential mental health benefit compared to controls with fewer mental health needs. While our study design does not allow inference of causality, it supports the restorative hypothesis, offers a means to quantify the potential effect size of greenspace exposure, and suggests feasibility of this method to offer potentially more valid data relating environment to symptoms.

One advantage of the methods utilized in this approach is the potential for longitudinal analysis. While the impact of environment on the symptoms of schizophrenia remains an ongoing topic of active study, smartphone-derived estimates of actual environmental exposure offer new data previously inaccessible. Given the low cost and unobtrusive nature of this data capture, it is possible to apply these methods over years to trace the impact on greenspace and other environmental exposure (like pollution levels) to explore the harm-reducing and capacity-building theories of environment on psychosis outcomes. Although the ethical implications of capturing such personal data for extended periods of time cannot be ignored, with the proper protections in place, the benefit of understanding the role of environment on the onset and trajectory of psychotic illness offers vast potential for improving patient outcomes.

Results indicating that higher greenspace is associated with lower symptom burden suggest several policy implications. Prior work from our team [22] and the current literature note that most people at risk for or with psychosis live in poor environments with less greenspace exposure. Given that it is now possible to measure the effect size of greenspace exposure on symptoms for individual participants, building an evidence-based case for the detrimental impact of certain environmental factors on mental health is now feasible. Future research following patients longitudinally as they move between neighborhoods with high and low greenspace exposure may provide the causal data needed to justify acting on results. In addition, while existing evidence of functional and structural changes associated with greenspace may relate to our results of symptom improvements, the evidence, especially in schizophrenia, is very limited. Our methodology can help better understand neurobiological mechanisms of action around greenspace exposure and mental health benefits as smartphones can support wearable EEG devices and smartphone sensor data may even inform brain connectivity [23].

Several limitations must be considered in interpreting our results. The study cohort was limited to both individuals with schizophrenia and healthy controls located in the metro Boston area and thus may not be generalizable. In addition, age, race, and education were not matched between SZ and HC, potentially introducing confounding variables. GPS accuracy from smartphones varied and we only included data with an accuracy threshold of 50 meters in the final analysis. NDVI is a commonly used measure of greenspace quality but may be less generalizable to drier climates like the American Southwest. Additionally, we were unable to classify the quality of greenspace exposure from satellite measurements. Also, the results of our paper are derived from group effects and results may be subject to ecological fallacy. Finally, our study made assumptions on the timeframes for environmental exposure and it is possible for result to differ with new timeframes.

## Conclusions

Our analysis of longitudinal GPS data from individuals in the greater Boston area revealed positive associations between time spent in higher greenspace areas and improved symptoms of depression, anxiety, and psychosis in individuals with schizophrenia. With limited studies reporting the effect on greenspace on symptomatology in serious mental illness, we hope that this paper reveals the potential of using smartphone sensing to capture and analyze such data, as well as the potential positive effects of greenspace in an urban environment. We recommend future research on more of an individual level to further elucidate the personal impact of one’s environment.
